# Influence of Post-UV/Ozone Treatment of Ultrasonic-Sprayed Zirconium Oxide Dielectric Films for a Low-Temperature Oxide Thin Film Transistor

**DOI:** 10.3390/ma13010006

**Published:** 2019-12-18

**Authors:** Abayomi Titilope Oluwabi, Diana Gaspar, Atanas Katerski, Arvo Mere, Malle Krunks, Luis Pereira, Ilona Oja Acik

**Affiliations:** 1 Laboratory of Thin Film Chemical Technologies, Department of Materials and Environmental Technology, Tallinn University of Technology, Ehitajate tee 5, 19086 Tallinn, Estonia; 2 i3N/CENIMAT, Department of Materials Science School of Science and Technology, FCT-NOVA, Universidade NOVA de Lisboa and CEMOP/UNINOVA, Campus de Caparica, 2829-516 Caparica, Portugal

**Keywords:** spray pyrolysis, low-temperature, zirconium oxide, Indium-Gallium-Zinc-Oxide, UV-ozone, high-κ dielectrics, thin film transistor

## Abstract

Solution-processed metal oxides require a great deal of thermal budget in order to achieve the desired film properties. Here, we show that the deposition temperature of sprayed zirconium oxide (ZrO_x_) thin film can be lowered by exposing the film surface to an ultraviolet (UV) ozone treatment at room temperature. Atomic force microscopy reveals a smooth and uniform film with the root mean square roughness reduced from ~ 0.63 nm (UVO-O) to ~ 0.28 nm (UVO-120) in the UV–ozone treated ZrO_x_ films. X-ray photoelectron spectroscopy analysis indicates the formation of a Zr–O network on the surface film, and oxygen vacancy is reduced in the ZrO_x_ lattice by increasing the UV–ozone treatment time. The leakage current density in Al/ZrOx/p-Si structure was reduced by three orders of magnitude by increasing the UV-ozone exposure time, while the capacitance was in the range 290–266 nF/cm^2^, corresponding to a relative permittivity (k) in the range 5.8–6.6 at 1 kHz. An indium gallium zinc oxide (IGZO)-based thin film transistor, employing a UV-treated ZrO_x_ gate dielectric deposited at 200 °C, exhibits negligible hysteresis, an I_on_/I_off_ ratio of 10^4^, a saturation mobility of 8.4 cm^2^ V^−1^S^−1^, a subthreshold slope of 0.21 V.dec^−1^, and a V_on_ of 0.02 V. These results demonstrate the potentiality of low-temperature sprayed amorphous ZrO_x_ to be applied as a dielectric in flexible and low-power-consumption oxide electronics.

## 1. Introduction

Zirconium oxide (ZrO_x_) has gained a large amount of attention in different applications such as thin film transistors (TFT) [1,2], sensors [3,4], display technology [1], and memory technology [5,6] due to its unique thermal stability, optical, and electronic properties. Additionally, in TFT applications, ZrO_x_ has been employed as a plausible replacement for the silicon oxide dielectric layer, owing to its high permittivity (κ) (~25), and wide bandgap (5.1–7.8 eV) [7,8,9]. However, the production of ZrO_x_ dielectrics by a wet chemical process is still slow because of high processing temperature (above 400 °C), arising from the need to decompose the organic moiety from the film’s matrix, which in turn increases the thermal budget [10,11].

Different authors have reported on the solution-processing technologies that can be used to produce ZrO_x_ dielectric films and the need for post deposition heat treatment in order to achieve a good-quality film that will yield promising electrical performance in TFTs [12,13,14,15]. For instance, according to Park et al., a ZrO_x_ dielectric was synthesized by adding hydrogen peroxide, and the fabricated dielectric film was tested as a TFT, which demonstrated a low leakage current with high breakdown strength (3.4 MV/cm) after film treatment at 350 °C [12]. Lee et al. [13] fabricated a solution-processed ZrO_x_ TFT on a glass substrate; however, the desired carrier mobility (~25 cm^2^/Vs) was achieved at a high annealing temperature of 500 °C. Ha and co-workers [14] employed solution-processed ZrO_x_ as a gate dielectric layer of Zinc Tin Oxide (ZTO)-TFTs, which demonstrated a low operating voltage (<5 V) and high channel carrier concentration, but the optimized annealing temperature of the ZrO_x_ dielectric film was as high as 500 °C. Oja et.al [16], Juma et al. [17], and Oluwabi et al. [18,19] have deposited metal oxide films by spray pyrolysis; in light of their results, the desired morphology and electrical properties were attained after annealing at temperatures above 700 °C. Also, Morvillo et.al [20] reported that annealing does not only influence the performance of metal oxide films but also influences electronic changes in their underlying substrate (e.g. ITO), which eventually makes the optimization process very challenging. 

In recent years, different approaches have been reported regarding material selection and curing conditions that can reduce the processing temperature (<250 °C) of solution-processed metal oxide films [21,22,23,24]. These approaches can be grouped into two groups: (1) chemical methods that deal with the chemistry of the precursor solution to facilitate a low external temperature [25]—for instance, in combustion synthesis [10,26,27]—and (2) annealing methods that use alternative energy sources or mediated annealing conditions to reduce the processing temperature of metal oxide thin films [25]—examples of this approach are vapour, photo, and vacuum annealing [28]. 

Among the annealing methods, photo-assisted annealing such as UV, laser, and pulsed light are potential alternatives to traditional high-thermal annealing because an adequate amount of light energy can directly illuminate the surface of the film. Kim et al. proposed an effective way to fabricate solution-processed metal oxide films using deep ultraviolet (DUV) irradiation at 150 °C [29]. Although the approach was highly efficient, the damage caused by such UV equipment may render it unattractive for production. Therefore, it is of great significance to develop a simple route to fabricate high-quality dielectrics at low temperature, as it would be suitable as a gate-dielectric layer in TFT application.

Here, we present a systematic study of the effect of the UV–ozone (UVO) treatment of s solution-processed ZrO_x_ dielectric by the ultrasonic spray pyrolysis (USP) method; the influence of UVO treatment was investigated with respect to the film’s morphology and electrical properties, while the optimized ZrO_x_ thin film was tested in indium gallium zinc oxide (IGZO)-based TFT devices. It is essential to point out that UVO treatment is newly introduced for sprayed ZrO_x_ dielectric films and will be informative for future studies, opening the possibility of depositing ZrO_x_ thin films onto flexible substrates for electronic applications. The deposition temperature reported in this study differs from a related work on the characterization of ZrO_x_ deposited by the wet chemical method [24,30]. Most literature usually adopts thermal annealing plus UV treatment, whereas in this present work, the photochemical post-deposition treatment was done at room temperature. 

### Mechanism of UV-Ozone Irradiation

UVO irradiation has been widely studied owing to its broad applicability in different fields, and two regions of wavelengths have been significantly reported in the production of ozone. First, light at λ < 243 nm splits the atmospheric oxygen molecules, and secondly light at 240 < λ < 320 nm decomposes ozone molecules to oxygen free radicals (O), which effectively performs the oxidative treatment of the ZrO_x_ films. The chemical reactions involved when the atmospheric air is used for ozone production are [24,31] as follows:

O_2_ (air)
+ (λ < 243 nm)    →    2O (free radicals)

O + O_2_    
 →     O_3_ (Ozone)

O_3_
+ (240 nm < λ < 320 nm)    →   O_2_ +
O

O + O_3_      
→       2O_2_

## 2. Materials and Methods 

ZrO_x_ thin films of about 20 nm thickness were deposited (T_dep_) at 200 °C by the ultrasonic spray pyrolysis (USP) technique, which uses a nebulizer operated at 1.5 MHz. The nebulized precursor solution consisted of zirconium acetylacetate (Zr(acac)_4_) and methanol. The resulting aerosol was transported onto a heated p–Si-wafer with the aid of air as the carrier gas (flow rate; 3 L/min). After the deposition process, the UVO cleaning process was carried out using a commercially available UVO system (NOVASCAN PSD-series, from Novascan Tech Inc., Boone, NC, USA) with UV-light (184.9 nm and 253.7 nm) generated by a mercury vapor lamp. The UVO exposure time was varied at 30, 60, and 120 min, respectively. The corresponding sample data in figure are labelled as UVO-0, UVO-30, UVO-60, UVO-120 for 0, 30, 60, and 120 min of exposure time, respectively. 

The surface morphology of the ZrOx dielectric film was determined by the atomic force microscopy (AFM, from NT-MDT, S & L, Ireland) technique in a non-contact mode. All the scans were taken in air using the instrument NT-MDT solver 47 pro with a resolution in the range of 3 nm, and the investigated area was 2 µm × 2 µm per scan. A silicon cantilever was employed as a probe for the AFM image acquisition and connected to a resonator. AFM measurements were carried out on both the untreated and UV–ozone (UVO)-treated ZrOx thin films to investigate the surface morphology and root mean square (RMS) roughness. The RMS roughness was estimated using the Gwyddion software (Version 2.54, GNU, General public license). The wettability of the ZrO_x_ dielectric films was studied using a DSA 25-KRÜSS instrument (from Krüss GmbH, Hamburg, Germany). The contact angle (CA) of water on the film surface was measured at room temperature using the sessile drop fitting method. X-ray photoelectron spectroscopy (XPS) measurements were performed on a Kratos Axis Ultra DLD (delay line detector) spectrometer (from Kratos Analytical Ltd., Manchester, England) in conjunction with a 165 mm hemispherical electron energy analyzer. Analyses were carried out with a monochromatic Al Kα X-ray source (1486.6 eV) operating at 150 W. The XPS spectra were recorded using an aperture slot of 300 μm × 700 μm and a base pressure of 2 × 10^−9^ Torr. The spectrometer was configured to operate with a 20 eV pass energy and a 90° take-off angle from the surface. The spectra were calibrated using a C 1s core level peak centered at a binding energy of 285.0 eV. 

Al contact was made using Quorum K975X vacuum evaporator (from Quorum Tech. Ltd., East-sussex, England) on top of the ZrO_x_ film surface with a contact area of 1.7 mm^2^, giving an Al/ZrO_x_/p–Si structure. The crystalline p–Si wafer was contacted through an indium metal electrode. The I–V curves were measured by applying a DC bias voltage from −1 to 1 V, while impedance measurements were taken by applying an AC signal of amplitude 20 mV in the frequency range of 100 Hz–1 MHz using AUTOLAB PGSTAT30/2. 

The TFTs were produced in a staggered bottom-gate, top-contact structure by depositing AlO_x_ thin films onto p-type silicon substrates (1−10 Ωcm). The IGZO semiconductor film was sputtered onto the ZrO_x_ thin films via a shadow mask from a commercial 2:1:1 IGZO ceramic target (from LTS Chemical Inc., Orangeburg, NY, USA) by radiofrequency magnetron sputtering without intentional substrate heating in an AJA 1300-F system. The sputtering atmosphere included an Ar/O_2_ flow ratio of 14:2, a 0.3 Pa deposition pressure, a power density of 4.9 W cm^2^, and a deposition time of 13 min 30 s to obtain a 30 nm thickness [32]. 

Finally, source and drain aluminium electrodes (80 nm thick) were deposited by thermal evaporation through a shadow mask onto films, and the ratio between the channel length and width was 10. Thereafter, the IGZO TFTs with the ZrO_x_ gate dielectric produced by spray were annealed at 150 °C temperature for 1 h in air. The output and the transfer characteristics of the devices were obtained in both forward and backward sweeps recorded in ambient conditions inside a Faraday cage using a semiconductor parameter analyser (Agilent 4155C, from Santa Clara, CA, USA). 

## 3. Results and Discussion

### 3.1. Surface Morphology and Wettability of the ZrOx Gate Dielectric Film

**Figure 1:**
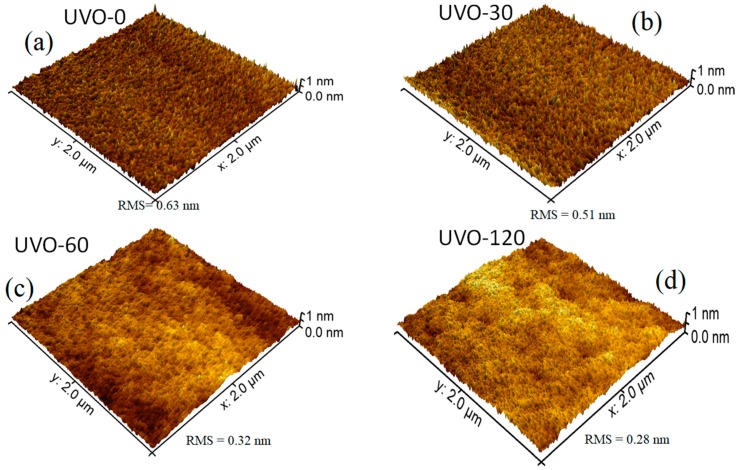
Atomic force microscopy (AFM) morphologies of (**a**) UV–ozone (UVO)-0 (untreated), (**b**) UVO-30, (**c**) UVO-60, and (**d**) UVO-120 treated ZrO_x_ dielectric thin films.

Figure 1 depicts the 3D AFM images (2 μm × 2 μm) for the ZrOx dielectric films at different UV-ozone exposure times (0–120 min), labelled as UVO-0, UVO-30, UVO-60, and UVO-120, respectively. Irrespective of the UVO treatment time, the ZrOx thin films demonstrated a plane surface morphology. The RMS roughnesses of the ZrO_x_ thin film at different UVO exposure times of 0, 30, 60, and 120 min were evaluated to be 0.63 nm, 0.51 nm, 0.32 nm, and 0.28 nm, respectively, indicating that the ZrOx thin films are smooth and that increasing the UVO exposure time reduces the surface roughness of the films. This reduction relative to UVO treatment is due to the removal of organic residue by the oxygen radicals produced during the UVO process, leaving the surface of the film very smooth with a low RMS roughness [11]. A smooth surface is a convenient requirement for the dielectric layer in TFTs because the surface roughness of a dielectric layer strongly influences the quality of the interface with the channel layer, which in turn plays a significant role in the operation of the TFT device [10].

**Figure 2:**
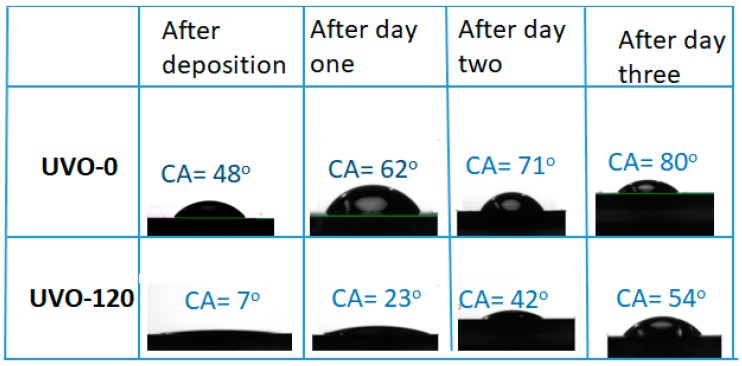
Images of the water contact angle (CA) measurements for both treated (UVO-120) and untreated (UVO-O) ZrO_x_ thin film and their corresponding contact angles after aging for three days.

The wettability of the ZrO_x_ dielectric surface was studied by measuring the water contact angle (CA), although the XRD pattern of the films indicated an amorphous structure (figure not shown). Figure 2 shows the droplet pictures alongside the mean CA values of water on both UVO-0 and UVO-120 treated ZrO_x_ films. It also shows the changes in CA values with aging. In the observed results, the UVO-120-treated ZrO_x_ dielectric is super-hydrophilic with a CA of 7°, while the UVO-0 film is hydrophilic with a CA of 48°.The intermediate treatment time (30 and 60 min) shows a CA value of 40°, which was 17° for UVO-30 and UVO-60, respectively. It is undoubtedly true that the UVO irradiation induces the presence of more OH-groups on the surface of the ZrO_x_ film due to the simultaneous conversion between the Zr–O–Zr and Zr–OH groups, thereby increasing the hydrophilicity of the ZrO_x_ film. A similar observation that supports this hypothesis was reported for ZrO_x_ thin films grown by dip-coating [33]; however, for ZrO_x_ dielectric films deposited by both sputtering [34] and electrochemical methods [35], a hydrophobic property was indicated. Gromyko et al. have also reported a difference in CA values for ZnO rods grown by both spray pyrolysis and electrodeposition methods [36].

Furthermore, after both samples were kept in a Petri-dish and allowed to age for three days, it was observed that the CA increased slightly in both UVO-120 and UVO-0 ZrO_x_ dielectric films. This indicates that the surface properties of the ZrO_x_ dielectric thin film can change owing to surface contamination from native carbon-containing species and that ZrO_x_ test samples should be kept in special conditions. This is because, during the treatment process, the oxygen radicals are produced to remove the organic residues present on the film’s surface, leaving the ZrO_x_ very active, and since test samples were not kept in any special conditions, this makes them vulnerable to native or environmental contaminants. 

### 3.2. XPS Characterization of ZrOx Gate Dielectric Film

**Figure 3:**
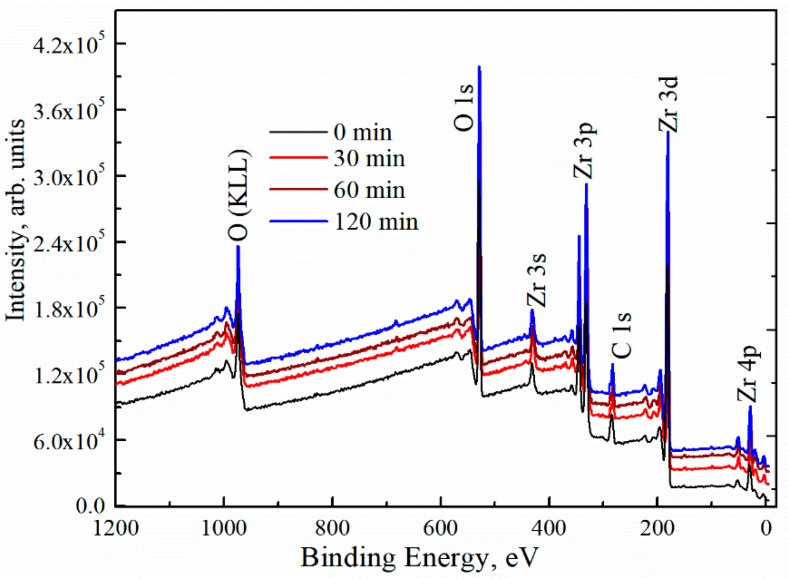
X-ray photoelectron spectroscopy (XPS) survey spectra of ZrOx thin films at different UV–ozone treatment times.

To study the surface composition of the deposited ZrOx thin films, XPS measurement was carried out. Figure 3 shows the survey XPS spectra for both the UVO (30–120)-treated and untreated ZrO_x_ dielectric films. The spectra showed zirconium features at Zr 3s (432.4 eV), Zr 3d (182.0, 184.4 eV), Zr 3p (346, 322 eV), and Zr 4p (30.8 eV) [37,38]. The C 1s peak of adventitious carbon is present at 284.6 eV for all films including the UVO-treated samples. Auger peaks for O (KLL) are also detected at the high-binding-energy region. The peak intensity of Zr 3d increases slightly with increasing UVO exposure time. In addition, the intensity of the O 1s peak was increased. Here, our discussion will be based on the O 1s and Zr 3d core levels, making a correlation between the UVO-0 and UVO-60 samples. 

**Figure 4:**
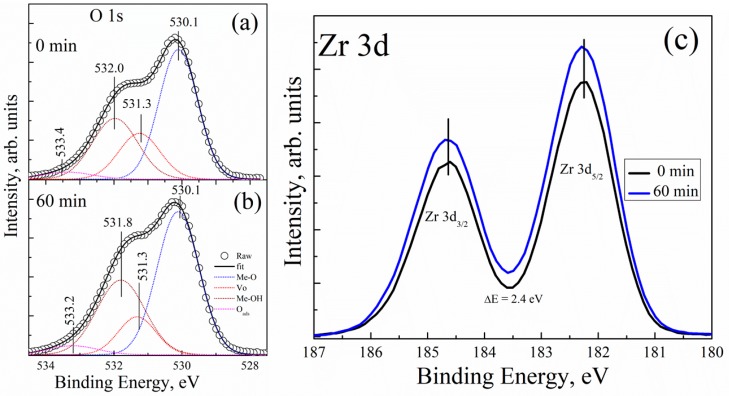
XPS spectra of the O 1s core level for (**a**) 0 minute and (**b**) 60 minutes of UVO treatment of the ZrOx dielectric film; the corresponding XPS spectra for the Zr 3d core level for the ZrO_x_ dielectric film is presented in (**c**).

Figure 4 shows the XPS spectra of the O 1s core level for two different UV-ozone treatment conditions. All the XPS spectra are asymmetric, and they were deconvoluted using Lorentzian–Gaussian (function pseudo-Voigt) distribution. Figure 4a displays the measured intensity of the O 1s core level of the UVO-0 sample; the peaks observed were fitted in four different components and centred at the binding energy (BE) values of 530.1, 531.3, 532.0, and 533.4 eV. The peak centred at 530.1 eV can be attributed to the BE of a well-bonded oxygen to zirconium (Me–O) in the ZrOx dielectric film lattice, while the peaks cantered at 531.3 imply the presence of an associated oxygen atom in the form of surface defects or vacancies in the film lattice (Vo). In addition, the peaks centred at 532.0 eV and 533.4 eV can be attributed to the oxygen in hydroxide-form (Me–OH) as a result of the high electronegativity of hydrogen atoms and the adsorbed oxygen (O_ads_), respectively [11,39]. Similarly, Figure 4b displays the measured intensity of the O 1s core level after a 60 minute UV-ozone treatment (UVO-60). The peaks on the XPS spectra are all located at BE and centred at BE values of 530.1, 531.3, 531.8, and 533.2 eV. The relatively weak peak component located at 533.2 eV is either due to oxygen connected with carbon or due to adsorbed oxygen in the form of moisture on the surface of the film [11,40,41]. This is certainly not connected to our samples, but to the environment (note that the films well sprayed directly on the Si-substrate were kept in a plastic box).

To resolve peak quantification properly, the peak ratios of all the components (MeO, Vo, −OH, H_2_O_ads_) found in the O 1s core level spectrum of UVO-0, UVO-30, UVO-60, and UVO-120 ZrO_x_ dielectric films were calculated from integrated areas of the O 1s spectrum using Scofield’s cross-sections, and their corresponding values are summarized in Table 1. We observe that, by increasing the UVO treatment time from 0 min to 120 min, the ZrO_x_ dielectric film demonstrated a slight increase in the [Me–OH]/[Me-O] component ratios, and a decrease in the [Vo]/[Me–O] ratio. It is confirmed that the UV–ozone treatment helps to eliminate the organic residues from the surface of the film, which makes our observation very reasonable, and a similar observation has been reported for solution-processed metal oxide films in [36,39,42]. The increase in the Me–OH component is an indication that the XPS study corresponds to the wettability study, suggesting that a high amount of –OH group at the film surface aided hydrophilicity in the UV-ozone treated ZrO_x_ films.

Figure 4c shows the corresponding XPS spectra of the Zr 3d core level for both UVO-0 and UVO-60 ZrO_x_ thin films. All the films showed the typical Zr 3d spectra with spin-orbit doublets (d_5/2_ = 182.3 eV, and d_3/2_ = 184.7 eV) separated by ~2.4 eV, which suggests the formation of ZrO_x_ thin films [17,43]. A similar BE has been reported for Zr 3d in our previous study on Zr-doped TiO_2_ films by spray pyrolysis [17].

According to the material characterisation section, an oxygen radical is generated by the UVO treatment to remove organic impurities, thereby effecting a change in the chemical properties of the ZrO_x_ film’s surface. This effect is evidently seen by the reduction in the donor defects (Vo) as well as an increase in the hydroxide group (Me-OH), actively changing the wettability and surface roughness of the ZrO_x_ dielectric film. The UVO treatment serves as a good insight to reduce the thermal budget of solution-processing techniques—especially spray pyrolysis. Furthermore, the amorphous structure of the ZrO_x_ dielectric film is essential to enhance the interface quality between different layers and to improve the electronic performance of the fabricated TFT device.

### 3.3. Electrical Characterization of ZrOx Capacitor

**Figure 5:**
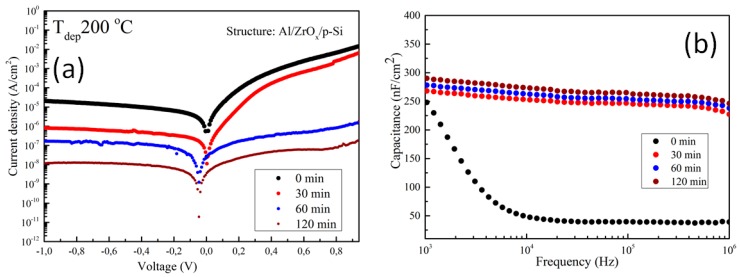
Electrical characterization of the metal insulator semiconductor (MIS) device made from ZrO_x_ dielectric: (**a**) current–voltage curve under positive and negative biases; and (**b**) capacitance–frequency dispersion curve in the range between 1 kHz and 1 MHz at different UV–ozone treatment times. The device has an Al/ZrOx/p–Si structure.

**Figure 6:**
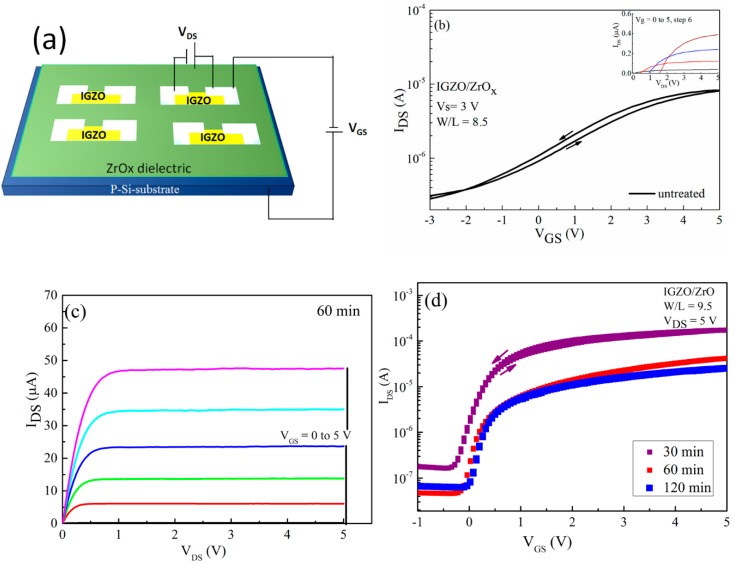
Thin film transistor (TFT) characteristics of indium gallium zinc oxide (IGZO)/ZrOx devices. (**a**) Schematic representation of device structure, (**b**) transfer and output (inset) characteristics of the devices with an untreated ZrOx gate dielectric, (**c**) output characteristic of a TFT-device with a treated ZrOx gate dielectric for 60 min, and (**d**) the transfer performance of the TFT devices with a treated ZrOx gate dielectric at different UV–ozone exposure times.

The electrical properties of the amorphous ZrO_x_ dielectric film was assessed by fabricating a metal insulator semiconductor (MIS) capacitor with the structure Al/ZrO_2_/p-Si. Figure 5a shows the plot of leakage current density–voltage (J-V) for ZrO_x_ dielectric films at different UVO treatment times. An asymmetric behavior can be seen due to the difference in the Schottky barrier height at the electrode interface. However, the leakage current density was calculated in the reverse bias regime, and it was found that the leakage current density in the UVO-0 ZrO_x_ dielectric film is ~ 2.0 × 10^−5^ A/cm^2^ at 1 V. A similar behavior has been reported for a ZrO_x_ dielectric deposited by atomic layer deposition (ALD) [44]. In contrast to the untreated sample, the UVO-treated samples demonstrated a remarkable reduction in leakage current density. Thus, by increasing the UVO treatment time to 30 min, the leakage current density was ~ 8.0 × 10^−7^ A/cm^2^, and a further increase in exposure time to 2 h yielded a leakage current density of ~ 1.0 × 10^−8^ A/cm^2^ at 1 V. It was reported that UV irradiation (λ > 185 nm) can produce hydroxyl radical (OH^.^) at a high quantum yield, which however aids the condensation reaction process of sol-gel metal oxide precursor films [25]. Therefore, it can be inferred that the reduction in the leakage current could be due to densification in the ZrO_x_ thin film, which occurred as a result of the longer UVO exposure time, increases the formation of metal–oxygen lattices and lowers the amount of oxygen defects on the surface of the ZrO_x_ thin film. A detailed explanation is given in the XPS study in Section 3.2. The zero-bias barrier heights of both the untreated and UVO-treated ZrOx dielectric were calculated by fitting the right part of Figure 6 into the following expression [42]:

(1) $$\emptyset_{B}=\frac{kT}{q}\ln\left(\frac{AA^{*}T^{2}}{I_{0}}\right)$$

where *A* is the effective area of the capacitor; A^*^ is the effective Richardson constant, which is equal to 36 Acm^−2^T^−2^ for ZrO_2_, assuming an electron effective mass 0.3 m_o_ for ZrO_x_; m_o_ is the free electron mass [30,44]; *k* is the Boltzmann constant; and $\emptyset_{B}$ is the Schottky barrier height.

The values of $\emptyset_{B}$ extracted from Equation (1) amounted to 0.76, 0.84, 1.04, and 1.09 eV for UVO-0, UVO-30, UVO-60, UVO-120 samples, respectively. The increase in $\emptyset_{B}$ could be due to surface changes at the ZrO_x_/electrode interface, which is caused by a change in surface energy due to UVO oxidative treatment. These values compared well with a similar report on a uniform and amorphous-ZrO_x_ dielectric film deposited on the native tungsten oxide surface by ALD [44,45]. The observed changes in the barrier height showed that the energy barrier at the electrode/dielectric interfaces is influenced by chemical changes at the surface of dielectric apart from the effect of the image charge build-up at the electrode. To fully understand the changes in the observed barrier height, the effect of charge traps in the dielectric located at the electrode interface of the capacitor constituent layers must be considered [46]. The obtained result is a good indicator of the potential applicability of UVO treatment in reducing the process temperature of solution-processed dielectric films by changes in the surface chemistry of the ZrOx dielectric layer.

To account for the dielectric properties of the deposited ZrO_x_ films, the capacitance–frequency (C–F) relation was measured at 0 V biased voltage. Figure 5b shows the C–F dispersion curve of ZrO_x_ capacitors measured at different UVO treatment times. The untreated ZrO_x_ dielectric demonstrated a high capacitance at the low frequency region, which suggests the contribution of ionic polarization [10]. On other hand, the UVO-treated samples exhibited a slight increase in capacitance (268, 272, and 290 nF/cm^2^ for UVO-30, UVO-60, and UVO-120 samples, respectively), which was stable in the high-frequency region. The result obtained from the UVO-treated samples revealed that, by increasing the UVO exposure time, the contributions from the interface or native oxide capacitance can be eliminated. This result concurs with the AFM and XPS results on the condensation and defect reduction from the surface of the UVO treated ZrO_x_ dielectric films.

According to the equation $\;C=\varepsilon_{0}\kappa A/d$, where *C* is capacitance, $\varepsilon_{0}\;$ is the permittivity of free space, κ is the relative permittivity, *A* is the contact area, and *d* is the ZrO_x_ film thickness (~ 20 nm), the relative permittivity (κ) of all the deposited ZrO_x_ films was calculated. As with the capacitance, the value of κ increases slightly from 5.8 to 6.6 with an increase in the UVO exposure time. It can be inferred from our previous study on ZrO_x_ dielectric films by spray pyrolysis that thermal annealing (~800 °C) was needed to obtain a κ value of 4.8 [19]. However, in this study, with UVO treatment, a κ value of 6.6 is obtained, thus indicating the advantage of UVO treatment in improving the properties of high-κ oxide dielectric films. 

Generally, we observed that the value of κ is smaller compared to the most anticipated theoretical κ value for ZrO_x_. This could be due to the influence of interfacial barriers in the film’s microstructure during deposition. Also, the sprayed deposited ZrO_x_ films are amorphous and inevitably contain pores because of the low deposition temperature. Nevertheless, this does not limit the performance of our film, as a similar value has been reported for a ZrO_x_ dielectric deposited by spin-coating in [2,11].

### 3.4. TFT Characterization of the Fabricated IGZO-Based Device

In order to ascertain the applicability of the deposited sprayed ZrO_x_ film in TFT, we fabricated a TFT with a bottom-gate–top-contact configuration, and the alignment between the channel and dielectric layer was patterned and staggered to reduce the probability of the source/drain infringing on the channel. Both the architecture and electrical performance of the TFT devices are shown in Figure 6, with the extracted electrical parameters presented in Table 2. All transistors which were fabricated are working and show a clear gate dependence corresponding to the n-type channel. The transfer characteristics of the IGZO based devices, which were measured in forward and backward sweeps for both the untreated and the UVO-treated ZrO_x_ gate dielectric, are shown in Figure 6b,d respectively. The device with the untreated gate dielectric showed a poor performance with a negative V_on_ of about −2 V, which means that the device was working in a depletion mode, and the ratio between the on current and off current (I_on_/I_off_) was very low at about 40 the leakage current (I_GS_) flowing through the gate was about 3.4 × 10^−2^ A. However, the device with a UVO-treated ZrO_x_ gate as its dielectric layer showed better electrical performance with negligible hysteresis, which later improved by increasing the UVO exposure time. The V_on_ changed from −0.3 V to 0.02 V when the UVO treatment time was increased from 30 to 120 min, respectively, indicating that the device changes its mode of operation from the depletion mode to enhancement mode. The positive shift in the device V_on_ could stem from the influence of the UV treatment influencing the surface potential of the ZrO_x_ dielectric. As the UV–ozone exposure time increases, a new chemical state is induced on the surface of the ZrO_x_ dielectric which is capable of effecting high electron trapping at the interface, particularly when a gate bias is applied to the device. Therefore, in order to compensate the charge, more mobile hole charges are induced, which explains the positively shifted threshold voltage of the TFT [47]. 

This result concurs with the wettability measurement as well as the Schottky barrier determination, demonstrating an increase in the energy barrier height caused by changes in the surface potential of the ZrO_x_ dielectric layer during the UV-ozone treatment. Furthermore, the magnitudes of the on–off current ratio, I_on_/I_off,_ are 1 × 10^3^, 0.4 × 10^4^, and 1 × 10^4^ when the UVO treatment time increases from 30, 60 to 120 min, respectively. The Figure 6c shows the typical output characteristic curve for the 60 min UVO-treated ZrO_x_ gate dielectric TFT. It can be seen that the UVO-treated TFTs exhibit typical n-type channel conduction behavior with a clear pinch-off voltage and current saturation. 

The fabricated TFT parameters, such as saturation mobility (µ_sat_), threshold voltage and sub-threshold slope, were extracted from the following equation [45] and are summarized in Table 2.

(2) $$I_{d}=\;\left(\frac{C_{ZrO}B\mu_{sat}}{2L}\right){\left(V_{G}-\;V_{T}\right)}^{2}$$

where $C_{ZrO}$ is the gate dielectric capacitance per unit area, B and L are the channel width and length, $V_{G}$ is the gate voltage, and $V_{T}$ is the threshold voltage, which was determined in the saturation regim by the fitting of the curve of Id12 versus $V_{G}$ and extrapolating the linear part to the $V_{G}$ axis. As expected, the devices with a UVO-treated ZrOx dielectric layer have better mobility, which increases from 2.9 to 8.4 cm^2^ V^−1^S^−1^ with increasing treatment time (from 30 to 120 min). 

The observed changes in mobility may be due to an increase in the capacitance of the ZrO_x_ gate dielectric layer when exposed to UVO treatment. According to Dong et al. [24] in their recent publication, there is the possibility of using UVO treatment to improve the device performance of InO/ZrO_x_ TFTs. Also, Carlos et al. [46] demonstrated the possibility of reducing the gate leakage current of IGZO/AlO_x_ TFT devices by using a very powerful UV lamp. The device demonstrated a positive shift in its threshold voltage (Vth) from −0.12 to 0.01 V, and a slight decrease in the sub-threshold slope (S) with increasing UVO treatment. The positive V_th_ indicated that the device can completely be switched off and can be turned on by a voltage as minimal as 0.01 V. In addition, the small S value extracted from the TFTs may be attributed to the large area capacitance at the ZrO_x_ gate dielectric layer and smoother surface due to the UVO cleaning of the layer, thereby improving the interface quality between IGZO and ZrO_x_ [11,39].

## 4. Conclusions

In summary, we have demonstrated the possibility of lowering the processing temperature of ultrasonically sprayed amorphous ZrO_x_ thin films by introducing UV–ozone post deposition treatment. It was confirmed by XPS and wettability measurement that by increasing the UVO exposure time, the surface of the sprayed ZrO_x_ films became less defective and hydrophilic, with a contact angle of 7°, indicating the removal of organic impurities associated with the precursor reagents from the surface of the film. The AFM result showed that the deposited ZrO_x_ film was smooth, and the surface roughness was reduced from 0.63 nm (UVO-0 film) to 0.28 nm (UVO-120 film). Finally, to demonstrate the electrical performance of the film, a MOS-capacitor was fabricated, and we observed a reduction in the leakage current density by three orders of magnitude by increasing the UV–ozone treatment time. The UVO treated ZrO_x_ capacitor attained desirable dielectric properties, such as a low leakage current density of 10^−8^ A/cm^2^, a capacitance of 290 nF/cm^2^ and relative permittivity of 6.6 (both at 1 kHz).

As a proof of concept, both untreated and UV–ozone post deposition-treated ZrO_x_ thin film were used as the gate dielectric in TFT. The fabricated TFT with the treated ZrOx films demonstrated an improved performance compared to a device produced with the untreated ZrOx dielectric thin film. The former operates in an enhancement mode (Von > 0), with low power consumption and a high saturation mobility of 8 cm^2^ V^-1^s^-1^. UV-ozone treatment is the key to developing a metal oxide film at low temperature by wet chemical techniques. This concept thus opens the potential application of ultrasonic spray technology in flexible electronics.

## Figures and Tables

**Table 1:** Binding energy of O 1s components and their corresponding ratios for UVO-0, UVO-30, UVO-60 and UVO-120 ZrO_x_ dielectric films.

Treatment Conditions	Binding Energy (eV)				Component Ratios	
	Me-O	Vo	Me-OH	OH _ads_	[Vo]/[Me-O]	[Me-OH]/[Me-O]
UVO-0	530.1	531.3	532.0	533.4	0.65	0.94
UVO-30	530.1	531.3	532.0	533.3	0.59	1.04
UVO-60	530.1	531.3	531.8	533.2	0.41	1.10
UVO-120	530.1	531.4	532.8	533.2	0.38	1.22

**Table 2:** Summary of the extracted TFT parameters for an average of ten IGZO/ZrOx devices at different UV–ozone treatment times.

Treatment Conditions	V_on_ (V)	I_on_/I_off_	V_th_ (V)	S (V.dec^−1^)	µ_sat_. cm^2^ V^−^^1^S^−^^1^	I_GS_ at V_GS_ = 5 V (A)
As-dep	−2.0 ± 1.0	~40	–	–	~0.02	~3.4 × 10^−2^
30 min	−0.3 ± 0.02	~1.0 × 10^3^	−0.12 ± 0.02	0.27 ± 0.02	2.9 ± 0.5	~7.4 × 10^−5^
60 min	−0.12 ± 0.1	~0.4 × 10^4^	0.02 ± 0.01	0.22 ± 0.01	7.0 ± 0.01	~2.3 × 10^−5^
120 min	0.02 ± 0.01	~1.0 ×10^4^	0.01 ± 0.005	0.21 ± 0.01	8.4 ± 0.01	~3.8 × 10^−7^
